# Comparison of the adverse event profiles of conventional and liposomal formulations of doxorubicin using the FDA adverse event reporting system

**DOI:** 10.1371/journal.pone.0185654

**Published:** 2017-09-27

**Authors:** Akiho Fukuda, Kohei Tahara, Yuuki Hane, Toshinobu Matsui, Sayaka Sasaoka, Haruna Hatahira, Yumi Motooka, Shiori Hasegawa, Misa Naganuma, Junko Abe, Satoshi Nakao, Hirofumi Takeuchi, Mitsuhiro Nakamura

**Affiliations:** 1 Laboratory of Drug Informatics, Gifu Pharmaceutical University, Gifu-shi, Gifu, Japan; 2 Laboratory of Pharmaceutical Engineering, Gifu Pharmaceutical University, Gifu University, Gifu-shi, Gifu, Japan; 3 Medical database Co., LTD, Shibuya-ku, Tokyo, Japan; Academia Sinica, TAIWAN

## Abstract

Doxorubicin (DOX) is an anthracycline widely used for the treatment of solid and hematological tumors. The aim of this study was to assess the adverse event profiles of conventional DOX and liposomal DOX. This is the first study to evaluate the effect of a liposomal formulation of DOX using spontaneous reporting system (SRS) databases. The SRS used was the US Food and Drug Administration Adverse Event Reporting System (FAERS). This study relied on definitions of preferred terms provided by the Medical Dictionary for Regulatory Activities (MedDRA) and the standardized MedDRA Queries (SMQ) database. We also calculated the reporting odds ratios (RORs) of suspected drugs (conventional DOX; PEGylated-liposome DOX; non-PEGylated-liposome DOX). The FAERS database contained 7,561,254 reports from January 2004 to December 2015. The number of reported AE cases for conventional DOX, PEGylated-liposome DOX, and non-PEGylated-liposome DOX was 5039, 3780, and 349, respectively. Conventional DOX and liposomal DOX have potential risks of causing myelosuppression, cardiotoxicity, alopecia, nausea, and vomiting, among other effects. The RORs (95% CI) from SMQ for *haematopoietic leucopenia* associated with conventional DOX, PEGylated-liposome DOX, and non-PEGylated-liposome DOX were 12.75 (11.89–13.68), 6.43 (5.81–7.13), and 14.73 (11.42–18.99), respectively. Liposomal DOX formulations were associated with lower RORs with regard to myelosuppression, cardiotoxicity, and alopecia than the conventional DOX was. The RORs (95% CI) for palmar-plantar erythrodysesthesia (PPE) associated with conventional DOX, PEGylated-liposome DOX, and non-PEGylated-liposome DOX were 6.56 (4.74–9.07), 64.77 (56.84–73.80), and 28.76 (15.77–52.45), respectively. This study is the first to evaluate the relationship between DOX liposomal formulations and their adverse event profiles. The results indicate that careful observation for PPE is recommended with the use of liposomal DOX, especially PEGylated-liposome DOX formulations.

## Introduction

Doxorubicin (DOX) is an anthracycline widely used for the treatment of solid and hematological tumors such as metastatic breast cancer, advanced ovarian cancer, acquired immunodeficiency syndrome-related Kaposi’s sarcoma, and acute myeloblastic leukemia. Conventional DOX administered as an intravenous bolus injection has been shown to cure select cases for over 30 years [1]. However, due to the insufficient tissue preference of conventional DOX, it is distributed to the entire body. Conventional DOX is toxic to most major organs and causes a number of serious side effects including cardiotoxicity, hematological disorders, and myelosuppression [1].

The most challenging issue in pharmaceutical engineering is the development of new drug delivery systems (DDSs), which enhance the therapeutic effects of drugs and reduce drug toxicity to organs. These systems usually work by either increasing drug concentration in tumor cells or decreasing the exposure of normal tissues to drugs. Liposomes are a novel type of DDS. Currently, several liposomal formulations of DOX (liposomal DOX) are available for clinical use [2].

These liposomal DOX preparations are divided into two categories: PEGylated liposome DOX (PEGylated-liposome DOX) [3,4] and non-PEGylated liposome DOX (non-PEGylated-liposome DOX) [5]. Liposomal formulations containing poly (ethylene glycol)(PEG)–modified, that is “PEGylated,” lipids exhibit an increased circulation time because of a reduced tendency to aggregate following intravenous injection [6,7]. Several studies have reported that patients treated with liposomal DOX demonstrate specific mucocutaneous adverse events (AEs) such as palmar-plantar erythrodysesthesia (PPE), which is also known as hand-foot syndrome. This AE limits the feasibility of antitumor chemotherapy with DOX and negatively affects a patient’s quality of life [8,9].

Spontaneous reporting systems (SRSs), wherein clinicians report their concerns about potential drug-induced AEs during their daily diagnostic assessments of patients, are useful for the detection of rare and severe AEs. The US Food and Drug Administration (FDA) Adverse Event Reporting System (FAERS) is the largest SRS in the world. It is recognized as the primary tool for pharmacovigilance that reflects the realities of clinical practice [10,11].

The aim of this study was to assess the AE profiles of conventional DOX and liposomal DOX. We also compared the results obtained for PEGylated-liposome DOX with those obtained for non-PEGylated-liposome DOX. To the best of our knowledge, this is the first study to evaluate the effects of liposomal formulations of DOX using the FAERS database.

## Materials and methods

Data from January 2004 to December 2015 in the FAERS database were downloaded from the FDA website (http://www.fda.gov/). The FAERS database structure complies with the international safety reporting guidelines (International Council on Harmonization, E2B). We then integrated the information obtained into a relational database using FileMaker Pro 13 software (FileMaker, Inc., Santa Clara, CA, USA). Next, we followed the recommendation by the FDA to use the most recent case numbers to identify duplicate reports for the same patient and excluded such data from the analysis.

Data on conventional DOX (Adriamycin^®^), PEGylated-liposome DOX [Doxil^®^ (Janssen Pharmaceuticals, Inc., Beerse, Belgium), Caelyx^®^ (Janssen Pharmaceuticals, Inc.), and LipoDox^®^ (Sun Pharmaceutical Industries Ltd., Mumbai, India)], and non-PEGylated-liposome DOX (Myocet^®^; Teva Pharmaceutical Industries Ltd., Petah Tikva, Israel) formulations were analyzed. Doxil^®^ and Caelyx^®^ are same liposomal DOX formulation. Drugs in the FAERS database are registered arbitrarily; for example, they may be registered as generic or brand names, or as abbreviations. DrugBank (The Metabolomics Innovation Centre, Canada, http://www.drugbank.ca/) is a reliable drug database used as a reference in pharmacovigilance analyses. Therefore, we used DrugBank (versions 3.0 and 4.0) as a source for batch conversion and compilation of drug names.

In the FAERS database, AEs are coded according to the terminology preferred by the Medical Dictionary for Regulatory Activities (MedDRA, http://www.meddra.org/; version 19.0). This study relied on definitions provided by MedDRA. We evaluated preferred terms (PTs) based on the top 30 AEs of conventional DOX. We also selected the following five characteristic AEs of DOX from the literature: *cardiomyopathy* (PT code: 10007636), *cardiotoxicity* (PT code: 10048610), *pulmonary embolism* (PT code: 10037377), *alopecia*, and PPE (Table 1) [1,3,4]. The standardized MedDRA Queries (SMQ) database is accepted and used in the analysis of SRSs [12–17]. Therefore, we used the SMQ for *haematopoietic leucopenia* (SMQ code: 20000030, containing 60 related PTs), *haematopoietic cytopenias affecting more than one type of blood cell* (SMQ code: 20000028, containing 27 related PTs), *haematopoietic erythropenia* (SMQ code: 20000029, containing 27 related PTs), *haematopoietic thrombocytopenia* (SMQ code: 20000031, containing 13 related PTs), and *interstitial lung disease* (SMQ code: 20000042, containing 60 related PTs).

**Table 1:** The reporting odds ratio (ROR) of conventional doxorubicin and liposomal doxorubicin in the FAERS database.

Adverse events		Total	Conventional Doxorubicin (conventional-DOX)			Pegylated Liposome Doxorubicin (PEGylated-liposome DOX)			Non-Pegylated Liposome Doxorubicin (non-PEGylated-liposome DOX)		
			Case ^b)^	ROR ^c)^	95%CI ^d)^	Case ^b)^	ROR ^c)^	95%CI ^d)^	Case ^b)^	ROR ^c)^	95%CI ^d)^
PT code ^a)^		6157897	5039			3780			349		
10016288	Febrile Neutropenia	19141	403	28.46	(25.68–31.54)	114	10.03	(8.32–12.09)	35	35.81	(25.25–50.80)
10037660	Pyrexia	123825	402	4.24	(3.82–4.69)	200	2.73	(2.36–3.14)	43	6.85	(4.98–9.43)
10029354	Neutropenia	33406	353	13.95	(12.51–15.55)	166	8.46	(7.24–9.89)	31	17.89	(12.37–25.87)
10028813	Nausea	267546	351	1.65	(1.48–1.84)	277	1.74	(1.54–1.97)	4	0.26	(0.10–0.68)
10013968	Dyspnoea	189849	331	2.21	(1.98–2.47)	254	2.27	(2.00–2.57)	7	0.64	(0.30–1.36)
10012735	Diarrhoea	174346	295	2.14	(1.90–2.40)	136	1.28	(1.08–1.52)	16	1.65	(1.00–2.72)
10047700	Vomiting	164691	286	2.19	(1.95–2.47)	192	1.95	(1.68–2.25)	6	0.64	(0.28–1.43)
10035664	Pneumonia	97081	275	3.61	(3.20–4.08)	154	2.65	(2.26–3.12)	8	1.46	(0.73–2.95)
-	Death	236778	271	1.42	(1.26–1.61)	236	1.67	(1.46–1.90)	5	0.36	(0.15–0.88)
-	Fatigue	213979	267	1.55	(1.37–1.76)	218	1.70	(1.48–1.95)	11	0.90	(0.50–1.65)
-	Pain	181319	263	1.82	(1.60–2.06)	151	1.37	(1.17–1.61)	3	0.29	(0.09–0.89)
10002034	Anaemia	74130	260	4.48	(3.95–5.07)	175	3.99	(3.43–4.65)	11	2.67	(1.47–4.87)
10043554	Thrombocytopenia	38176	237	7.95	(6.98–9.07)	133	5.86	(4.93–6.97)	9	4.24	(2.19–8.23)
10003549	Asthenia	130494	221	2.12	(1.85–2.43)	158	2.02	(1.72–2.36)	6	0.81	(0.36–1.81)
10031264	Osteonecrosis	14428	213	19.06	(16.60–21.88)	62	7.13	(5.54–9.16)	3	3.69	(1.18–11.51)
10040047	Sepsis	39218	212	6.88	(6.00–7.90)	79	3.33	(2.67–4.17)	8	3.66	(1.82–7.38)
-	Disease Progression	35325	201	7.24	(6.28–8.34)	153	7.34	(6.24–8.63)	12	6.17	(3.47–10.98)
10051398	Malignant Neoplasm Progression	20003	176	11.20	(9.63–13.02)	155	13.22	(11.25–15.53)	3	2.66	(0.85–8.29)
10003988	Back Pain	80619	171	2.65	(2.28–3.09)	131	2.71	(2.28–3.23)	5	1.10	(0.45–2.65)
-	Bone Disorder	9724	170	22.45	(19.24–26.19)	43	7.30	(5.40–9.87)	1	-^†^	-^†^
10029331	Neuropathy Peripheral	23664	169	9.05	(7.76–10.56)	111	7.87	(6.52–9.52)	0	-^†^	-^†^
-	Infection	38600	166	5.42	(4.64–6.33)	67	2.86	(2.25–3.65)	8	3.72	(1.85–7.50)
10033661	Pancytopenia	20256	163	10.20	(8.72–11.93)	119	9.90	(8.24–11.89)	6	5.30	(2.36–11.88)
10035598	Pleural Effusion	24089	161	8.45	(7.22–9.90)	98	6.80	(5.56–8.31)	2	1.47	(0.37–5.89)
10006002	Bone Pain	20459	157	9.71	(8.28–11.39)	32	2.56	(1.81–3.63)	1	-^†^	-^†^
10012174	Dehydration	54385	157	3.62	(3.08–4.24)	98	2.99	(2.45–3.66)	3	0.97	(0.31–3.03)
10003239	Arthralgia	118887	154	1.60	(1.36–1.88)	59	0.81	(0.62–1.04)	0	-^†^	-^†^
10028116	Mucosal Inflammation	8673	151	22.27	(18.92–26.22)	77	14.87	(11.85–18.65)	7	14.52	(6.87–30.70)
10000081	Abdominal Pain	82672	146	2.19	(1.86–2.59)	148	3.00	(2.54–3.53)	3	0.64	(0.20–1.99)
10002855	Anxiety	104278	145	1.72	(1.46–2.03)	58	0.90	(0.70–1.17)	1	-^†^	-^†^
10007636	Cardiomyopathy ^e)^	6807	103	19.13	(15.72–23.29)	21	5.06	(3.29–7.78)	0	-^†^	-^†^
10048610	Cardiotoxicity ^e)^	1673	33	24.73	(17.50–34.94)	9	8.82	(4.58–17.00)	1	-^†^	-^†^
10037377	Pulmonary embolism ^e)^	46729	78	2.06	(1.65–2.57)	65	2.29	(1.79–2.93)	7	2.68	(1.27–5.66)
-	Alopecia ^e)^	43514	79	2.24	(1.79–2.80)	29	1.09	(0.75–1.57)	2	0.81	(0.20–3.25)
-	Palmar-plantar erythrodysaesthesia syndrome ^e)^	6972	37	6.56	(4.74–9.07)	250	64.77	(56.84–73.80)	11	28.76	(15.77–52.45)
SMQ code ^f)^											
20000028	Haematopoietic cytopenias affecting more than one type of blood cell	51358	347	8.85	(7.93–9.87)	187	6.21	(5.36–7.19)	11	3.87	(2.12–7.06)
20000029	Haematopoietic erythropenia	127689	383	3.89	(3.51–4.32)	257	3.45	(3.04–3.92)	13	1.83	(1.05–3.18)
20000030	Haematopoietic leucopenia	114301	973	12.75	(11.89–13.68)	409	6.43	(5.81–7.13)	76	14.73	(11.42–18.99)
20000031	Haematopoietic thrombocytopenia	73937	335	5.88	(5.26–6.57)	208	4.80	(4.17–5.52)	11	2.68	(1.47–4.88)
20000042	Interstitial lung disease	49703	286	7.43	(6.59–8.38)	181	6.20	(5.34–7.20)	23	8.67	(5.68–13.24)

^a)^ Preferred term,

^b)^ Number of patients with adverse events,

^c)^ Reporting Odds Ratio,

^d)^ Confidence interval,

^e)^ Selected PTs based on characteristic adverse events associated with DOX,

^f)^ Standardized MedDRA Queries,

^†^Number of cases < 2.

**Fig 1:**
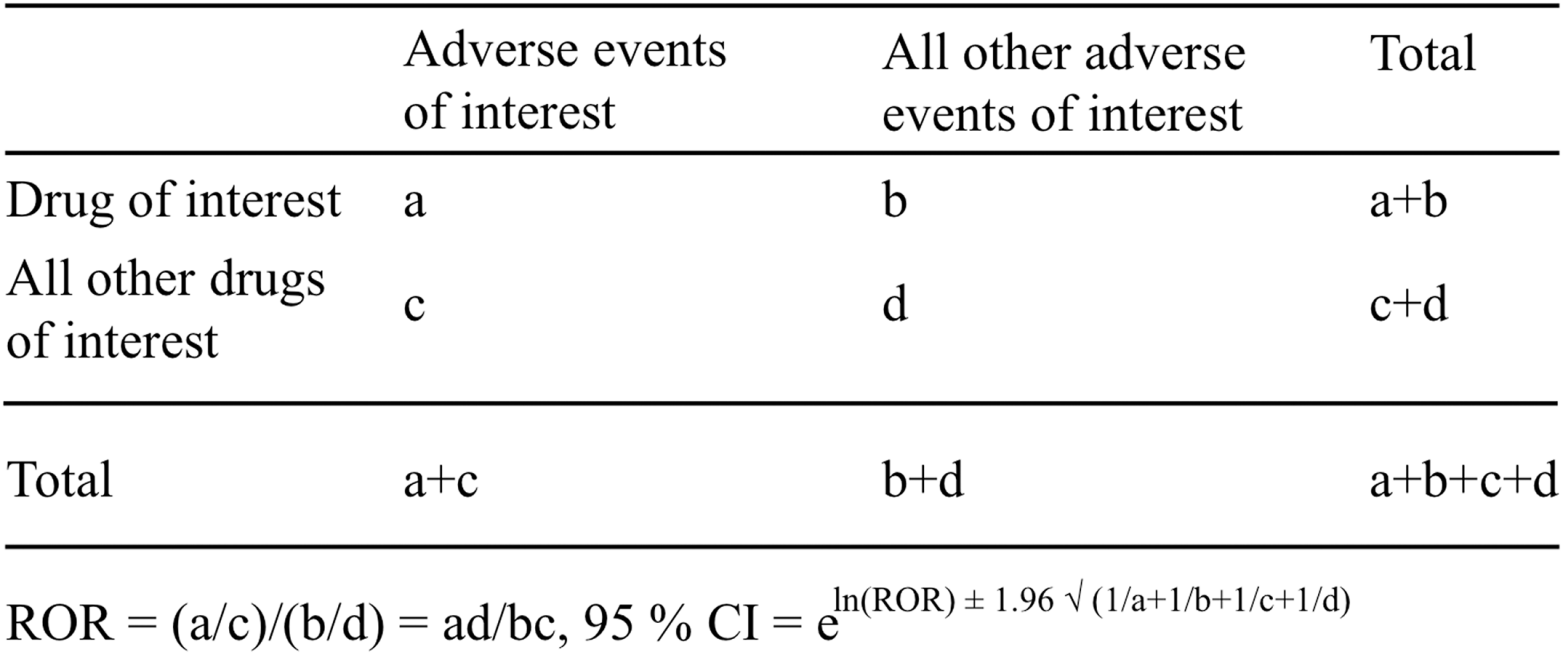
Two-by-two contingency table for analysis.

The effects of DOX were evaluated using the established pharmacovigilance index reporting odds ratio (ROR) [10,18]. Next, a two-by-two contingency table was constructed (Fig 1), and disproportional AEs and drug combinations were identified. A “case” was defined as a patient who reported an AE following the use of conventional DOX and liposomal DOX, whereas a “non case” was defined as a patient associated with all other events. ROR values were calculated as (a*d)/(b*c) and expressed as point estimates with a 95% confidence interval (CI). An event was considered significant when the lower limit of the 95% CI of the ROR was greater than 1 [18]. At least 2 cases were required to define a signal [10].

## Results

**Fig 2:**
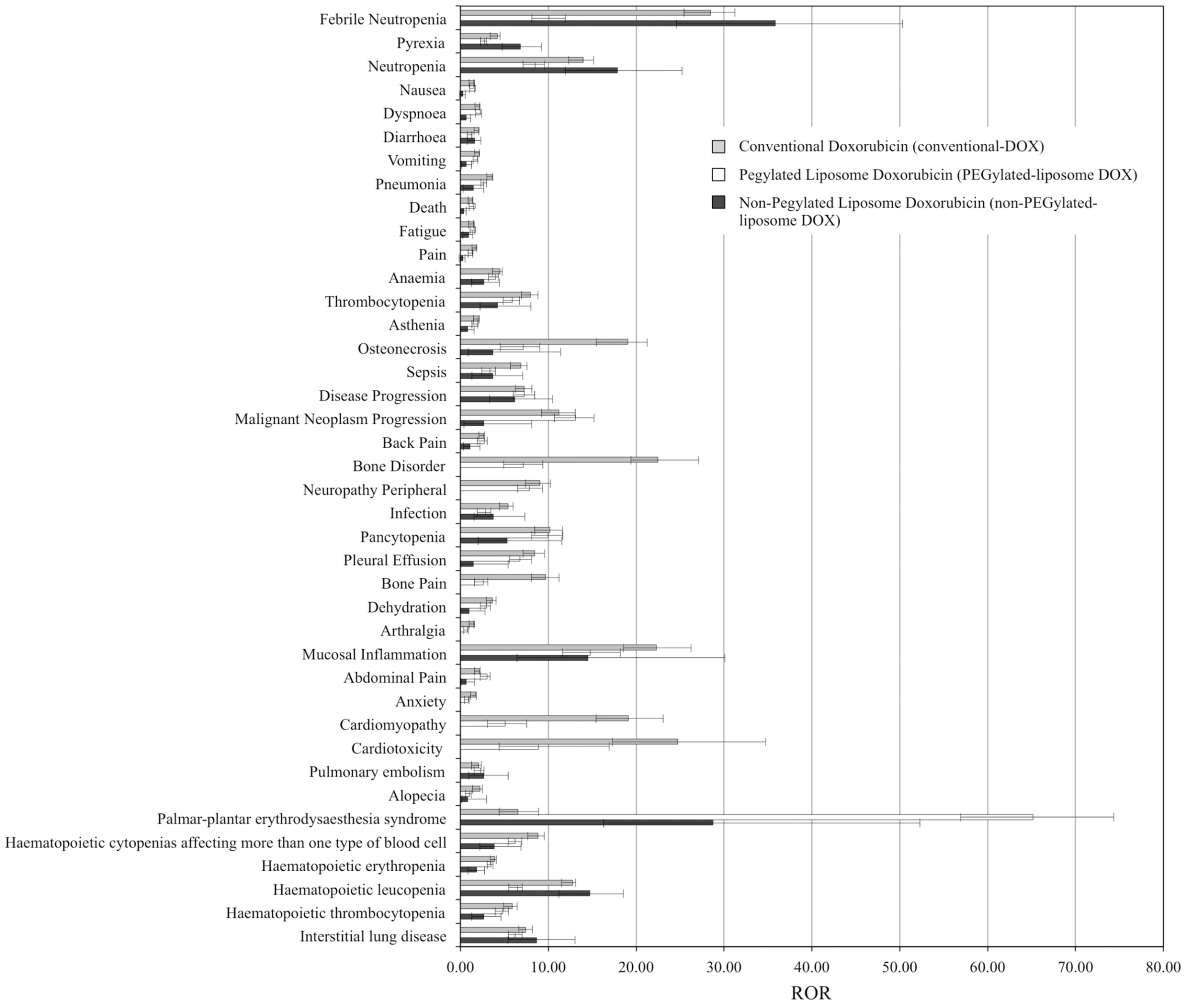
ROR for conventional DOX and liposomal DOX in the FAERS database.

The FAERS database contained 7,561,254 reports from January 2004 to December 2015. After excluding duplicate reports according to FDA recommendations, 6,157,897 reports were analyzed. The number of reported AE cases for conventional DOX, PEGylated-liposome DOX (Doxil^®^, Caelyx^®^, LipoDox^®^), and non-PEGylated-liposome DOX (Myocet^®^) was 5039, 3780, and 349, respectively (Table 1). The number of reported AE cases for Doxil^®^, Caelyx^®^, LipoDox^®^, and Myocet^®^ was 2641, 1121, 25, and 349, respectively. The RORs for conventional DOX and liposomal DOX (PEGylated-liposome DOX and non-PEGylated-liposome DOX) are summarized in Table 1 and Fig 2. The RORs (95% CI) for *febrile neutropenia* (PT: 10016288) associated with the use of conventional DOX, PEGylated-liposome DOX, and non-PEGylated-liposome DOX were 28.46 (25.68–31.54), 10.03 (8.32–12.09), and 35.81 (25.25–50.80), respectively, whereas the corresponding values for *cardiomyopathy* were 19.13 (15.72–23.29), 5.06 (3.29–7.78), and not applicable (number of cases < 2). Furthermore, the RORs (95% CI) for *cardiotoxicity* associated with the use of conventional DOX, PEGylated-liposome DOX, and non-PEGylated-liposome DOX were 24.73 (17.50–34.94), 8.82 (4.58–17.00), and not applicable (number of cases < 2), respectively, whereas the corresponding values for *alopecia* were 2.24 (1.79–2.80), 1.09 (0.75–1.57), and 0.81 (0.20–3.25), respectively. However, those for PPE were 6.56 (4.74–9.07), 64.77 (56.84–73.80), and 28.76 (15.77–52.45), respectively. The RORs (95% CI) of SMQ for *haematopoietic leucopenia* associated with the use of conventional DOX, PEGylated-liposome DOX, and non-PEGylated-liposome DOX were 12.75 (11.89–13.68), 6.43 (5.81–7.13), and 14.73 (11.42–18.99), respectively.

Table 1 lists the 30 PTs that were mostly used in reporting AEs. In addition, the PTs have been sorted by their reporting numbers. For conventional DOX, the most commonly reported AEs were febrile neutropenia, pyrexia, neutropenia, and nausea. The RORs for febrile neutropenia, bone disorder, mucosal inflammation, osteonecrosis, and neutropenia were highly ranked. For PEGylated-liposome DOX, the RORs for PPE, febrile neutropenia, and neutropenia were highly ranked. Furthermore, for non-PEG-liposome DOX, the RORs for PPE, febrile neutropenia, and mucosal inflammation were highly ranked. The RORs for alopecia and cardiotoxicity due to the use of PEGylated-liposome DOX and non-PEGylated-liposome DOX were low compared to those due to the use of conventional DOX. Moreover, the ROR of SMQ for *haematopoietic leucopenia* associated with the use of PEGylated-liposome DOX was lower than that associated with the use of conventional DOX. In addition, the ROR for PPE due to treatment with PEGylated-liposome DOX was higher than that due to treatment with non-PEGylated-liposome DOX.

## Discussion

The results of the present study suggest that conventional DOX and liposomal DOX have potential risks of causing myelosuppression, cardiotoxicity, alopecia, nausea, and vomiting, which are side effects of DOX that have been reported in clinical practice. Alopecia, severe nausea and vomiting, and mucositis are AEs that may limit therapy with DOX. Liposomal DOX had lower RORs for myelosuppression, cardiotoxicity, and alopecia than conventional DOX did. In a clinical trial on metastatic breast cancer, liposomal DOX had a better safety profile for cardiotoxicity, neutropenia, vomiting, and alopecia than conventional DOX did; however, the two formulations demonstrated equivalent efficacy [19].

There are different profiles of AEs for conventional DOX and liposomal DOX in the FAERS database. The ROR for PPE in patients who received liposomal DOX was higher than that in those who received conventional DOX. In addition, it has been reported that PPE, stomatitis, and mucositis are more often associated with the use of PEGylated-liposome DOX than with the use of conventional DOX [19]. PPE causes redness, swelling, and pain. The pathophysiology of PPE following treatment with liposomal DOX has not been fully elucidated. It has been proposed that PEGylated-liposome DOX may be excreted in sweat from the eccrine sweat glands, which are mostly found in the palms and on the feet, where they produce sweat continuously. Since PEGylated-liposome DOX has a hydrophilic coating, it is carried to the skin surface via sweating. Moreover, a high concentration of PEGylated-liposome DOX was detected in the eccrine glands in a previous study [20]. From the skin surface, sweat containing DOX may reach the stratum corneum, through which DOX may enter and reach deeper skin layers and react with epidermal cells [20]. Although the incidence of severe PPE associated with the use of DOX is low, PPE can have a significant impact on quality of life. When administering PEGylated-liposome DOX to patients, early interventions such as dose reduction and drug withdrawal may be required.

Non-PEGylated-liposome DOX lacks a PEGylated membrane around the DOX-carrying liposomes, which gives the formulation some advantages, such as specificity and moderate toxicity, over other types of liposomal DOX formulations [2,21]. In the present study, the ROR for PPE resulting from treatment with non-PEGylated-liposome DOX was lesser than that resulting from treatment with PEGylated-liposome DOX. The ROR signal for alopecia was not detected for the non-PEGylated-liposome DOX.

For conventional DOX, the lower limit of 95% CI of ROR for cardiotoxicity was greater than 1 in the present study. In addition, high cumulative doses of DOX increase the cardiotoxicity of DOX. The results of a retrospective analysis of FAERS by Wittayanukorn et al., in which cardiotoxicity occurring during chemotherapy was examined, showed increases in the ROR values for DOX [22]. The number of reports and RORs for bone disorder and osteonecrosis were relatively high. However, we do not have a conclusive explanation for these data. More detailed analyses focusing on these factors will be performed in future investigations.

The lipid components of Doxil^®^/Caelyx^®^ are composed of hydrogenated soy of L-α-phosphatidylcholine (HSPC):cholesterol:PEG 2000-DSPE [PEGylated phospholipid (DSPE-PEG2000): 1,2-distearoyl-sn-glycero-3-phosphoethanolamine-N-[methoxy(polyethyleneglycol)-2000]] (56:39:5 molar ratio) [6,7]. LipoDox^®^ is composed of 1,2-distearoyl-sn-glycero-3-phosphocholine (DSPC):cholesterol:PEG 2000-DSPE (56:39:5 molar ratio) [6,7]. Myocet^®^ is composed of egg phosphocholine (EPC): cholesterol (55:45 molar ratio). Different lipid components may affect the safety profiles because of various carrier parameters: drug release rate from the liposome, residence time in the body, and interaction with cells.

PEGylated-liposome DOX demonstrates improved pharmacokinetics and biodistribution of DOX. In addition, it minimizes toxicity by accumulating in target tissues. The results of a pharmacokinetic study on PEGylated-liposome DOX showed that AEs, dose, and C_max_ correlated with the severities of stomatitis and leukocyte nadir, whereas the severity of PPE is significantly correlated with the half-life of DOX [23]. PEGylated-liposome DOX is a long-circulating PEGylated liposome in which DOX hydrochloride is encapsulated [24,25]. It accumulates in tumor tissues by passive targeting which is known as enhanced permeation and retention (EPR) [24]. The elimination half-lives and area under the plasma concentration-time curve (AUC) of DOX following treatment with conventional DOX (free DOX), PEGylated-liposome DOX, and non-PEGylated-liposome DOX were 0.2 h and 4 μg h/mL, 55 h and 900 μg h/mL, and 2.5 h and 45 μg h/mL, respectively [25]. Doxil^®^/Caelyx^®^ has a much longer circulation time due to the steric barrier provided by the surface-grafted PEG, which increases the amounts of DOX being delivered to the targeted tissue. This is advantageous for the treatment of skin-localized cancers such as Kaposi’s sarcoma but not for PPE [6]. Clinical data have indicated that patients might develop PPE after receiving multiple injections of PEGylated-liposome DOX [26,27]. This could explain why high ROR values were obtained for PPE. LipoDox^®^ was approved as a generic liposomal preparation of DOX for the Doxil^®^ with “AB” equivalency rating by the FDA. In an animal microdialysis study, total DOX exposure, as assessed based on AUC, was 2.5–2.9-fold higher for Doxil^®^ than for LipoDox^®^ [28]. A prospective clinical comparison might be required to determine the equivalency between Doxil^®^ and LipoDox^®^ [28].

Non-PEGylated-liposome DOX formulation such as Myocet^®^ is an EPC–cholesterol formulation. It releases DOX rapidly and has a much shorter circulation time in blood than PEGylated-liposome DOX does. It has been clinically shown to reduce DOX-induced cardiotoxicity and gastrotoxicity [6,7].

Moreover, the particle sizes of PEGylated-liposome DOX and non-PEGylated-liposome DOX are about 100 nm and 190 nm, respectively. It has been reported that the elimination half-life of liposomal DOX decreases with increasing size, negative charge density, and fluidity in the bilayer [2].

Immunogenicity of PEG may affect the AE profiles of liposomal DOX. Anti-PEG IgM elicited by injection of liposomes is involved in the enhanced blood clearance of a subsequent dose of PEGylated liposomes [29–31]. PEGylated liposomes lose their long-circulating characteristic upon repeated injection at certain intervals, referred to as the “accelerated blood clearance (ABC) phenomenon.” PEGylated-liposome DOX (Doxil^®^, Caelyx^®^, and LipoDox^®^) might be subject to immunogenicity and non-PEGylated-liposome DOX (Myocet^®^) might not be subject to. However, it was difficult to interpret the AE profile from our data in terms of immunogenicity.

A meta-analysis of the published randomized trials evaluated the AEs of liposomal DOX and anthracyclines and quantified the relative safety profile [32]. Liposomal DOX and PEGylated-liposome DOX showed favorable toxicity profiles with better cardiac safety and less myelosuppression and alopecia [32]. The meta-analysis did not show significant differences in PPE. On the contrary, our results demonstrated the differences in PPE. The data from the FAERS did not correspond with those that were analyzed in the meta-analysis. We do not have a conclusive explanation. Concato et al. reported that the results of well-designed observational studies do not systematically overestimate the magnitude of the effects of treatment as compared with randomized controlled trials on the same topic [33]. Our study is an observational study using SRS. Information from our dataset might be considered of complementary value.

ROR is a pharmacovigilance index that is clear and easy to understand (Fig 1). However, it is different from “odds ratio”, which is commonly used in epidemiological studies. The FAERS database is subject to various biases such as under-reporting, over-reporting, exclusion of healthy individuals, and confounding by comorbidities [18]. The data subsetting strategy may help to mitigate the effect of confounding factors and bias on signal detection by limiting the analysis to a population of patients that are thought to share common risk factors and diseases [15,16,34–36].

In particular, the dosage and treatment interval of three forms of DOX (conventional DOX, PEGylated-liposome DOX or non-PEGylated-liposome DOX) may have great impact on the AE profiles. However, the FAERS database is an SRS; therefore, detailed patient information is not included. Therefore, it was difficult to obtain and evaluate the accurate dosage and duration of the drugs from the FAERS database. Since the information on onset date for each drug was not recorded in the FAERS, time-to-onset could not be calculated from the start of a subject’s first prescription to the occurrence of the AEs. We did not analyze this further; however, detailed analysis of the effect of administration methods is warranted.

Basically, the higher the ROR value, the higher the risk of an AE. In absolute terms, ROR indicates an increased risk of AE reporting and not a risk of AE occurrence. In general, ROR is inapplicable to inferences of comparative strengths of causality and only offers a rough indication of signal strength. We must, therefore, highlight this profound limitation of the FAERS database.

Despite the inherent limitations of the SRS data, the results we obtained regarding the association between suspected DOX formulations are in agreement with the results of previous studies. This study is the first to evaluate the relationship between liposomal formulations of DOX and their AE profiles based on real clinical settings. The data we have reported is beneficial to pharmaceutical researchers and clinicians. In addition, the information we have provided will be potentially useful for improving chemotherapy with DOX.

## Conclusion

This study is the first to evaluate the relationship between liposomal DOX formulations and their AE profiles. The results of this study show that the use of liposomal DOX is associated with lower RORs for myelosuppression, cardiotoxicity, and alopecia than the use of conventional DOX is. Moreover, the ROR for PPE in patients receiving liposomal DOX is higher compared to that in patients receiving conventional DOX. Furthermore, the ROR for non-PEGylated-liposome DOX was lesser than that for PEGylated-liposome DOX. The results indicate that careful observation for PPE is necessary in patients being treated with DOX, especially the PEGylated-liposome DOX formulation.
